# Spotlight: Visualization of Moiré Quantum Phenomena in Transition Metal Dichalcogenide with Scanning Tunneling Microscopy

**DOI:** 10.1021/acsaelm.3c01328

**Published:** 2024-02-01

**Authors:** Hao Zhou, Kangkai Liang, Liya Bi, Yueqing Shi, Zihao Wang, Shaowei Li

**Affiliations:** ‡ Department of Chemistry and Biochemistry, University of California, San Diego, La Jolla, California 92093-0309, United States; § Program in Materials Science and Engineering, University of California, San Diego, La Jolla, California 92093-0418, United States; ⊥ School of Physics, Nankai University, Tianjin 300071, China

**Keywords:** moiré superlattice, transition metal dichalcogenide, scanning tunneling microscopy, strongly correlated phases, moiré exciton

## Abstract

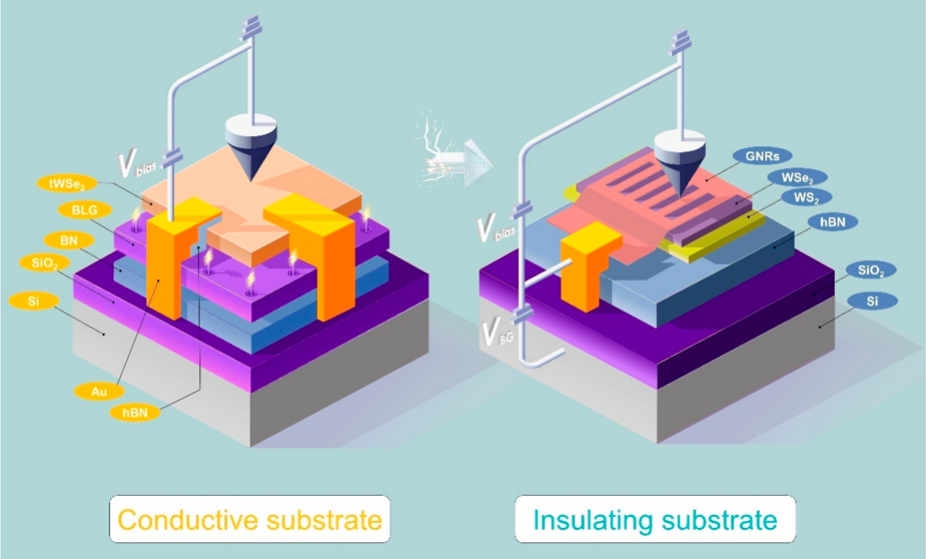

Transition metal dichalcogenide (TMD) moiré superlattices have emerged as a significant area of study in condensed matter physics. Thanks to their superior optical properties, tunable electronic band structure, strong Coulomb interactions, and quenched electron kinetic energy, they offer exciting avenues to explore correlated quantum phenomena, topological properties, and light–matter interactions. In recent years, scanning tunneling microscopy (STM) has made significant impacts on the study of these fields by enabling intrinsic surface visualization and spectroscopic measurements with unprecedented atomic scale detail. Here, we spotlight the key findings and innovative developments in imaging and characterization of TMD heterostructures via STM, from its initial implementation on the in situ grown sample to the latest photocurrent tunneling microscopy. The evolution in sample design, progressing from a conductive to an insulating substrate, has not only expanded our control over TMD moiré superlattices but also promoted an understanding of their structures and strongly correlated properties, such as the structural reconstruction and formation of generalized two-dimensional Wigner crystal states. In addition to highlighting recent advancements, we outline upcoming challenges, suggest the direction of future research, and advocate for the versatile use of STM to further comprehend and manipulate the quantum dynamics in TMD moiré superlattices.

## 1 Introduction

Transition metal dichalcogenides (TMDs) have emerged as a promising and dynamic area of research within condensed matter physics, primarily due to their two-dimensional nature in combination with remarkable magnetic,^1−3^ optical,^4−6^ and electronic^7−9^ properties that hold the potential to revolutionize various technological applications and fundamentally enhance our understanding of the material. TMDs are characterized by the general formula MX_2_ where M represents a transition metal and X denotes chalcogen. The weak interlayer van der Waals interaction allows the easy preparation of monolayer TMDs by mechanical exfoliation,^10^ as well as the fabrication of heterogeneous junctions by superposing TMD layers on top of each other.^11^ By controlling the applied material, stacking order, or twist angle, the properties of the heterostructure can be engineered with unprecedented tunability.^12,13^ Specifically, a moiré superlattice can form when the structures of adjacent layers are close to commensuration, which creates a long periodicity due to the small lattice mismatch. This could be formed either by twisting layers of the same material at an appropriate angle or by aligning two different materials with a small lattice difference. In the momentum space, the superlattice defines another Brillouin zone with a smaller reciprocal lattice, which folds the parabolic band structure defined by the atomic lattice into a sequence of new bands characterized by remarkably narrow bandwidths, commonly referred to as moiré flat bands.^14−16^ Similar to their graphene counterparts, these TMD moiré superlattices have undergone extensive investigation in both theoretical studies and experiments primarily because of the diverse quantum phenomena they facilitate.

While graphene has garnered significant attention as a two-dimensional material for its high accessibility,^17^ high symmetricity,^18^ and superior carrier mobility,^19^ TMD moiré systems offer unique advantages. First, compared to graphene, which consists solely of carbon atoms, TMDs provide a versatile range of materials achieved by substituting either the transition metal or the chalcogen atoms within the same group. Numerous TMDs, such as MoS_2_ and WSe_2_, share similar atomic structures but possess distinctive electronic properties.^20^ This abundance of options in building blocks offers a multitude of choices for crafting functional devices tailored to specific applications. Second, the natural existence of a bandgap close to the visible range in most TMDs also makes them suitable for a broad range of optoelectronic applications.^5,21^ Moreover, the incorporation of heavy transition metals into the structural framework significantly enhances spin–orbit coupling, holding promise for various spintronic applications.^3,22^ Most strikingly, the reduced dimension, together with limited dielectric screening from the surrounding, leads to a substantial amplification of the Coulomb interaction.^23−25^ Likewise, the creation of ultranarrow moiré flat bands can effectively modulate the charge carrier behaviors in them. The kinetic energy of the electrons residing in these flat bands becomes significantly restricted.^26,27^ Consequently, the Coulomb interaction frequently takes precedence in electron interactions, leading to the emergence of a sequence of highly correlated quantum phenomena including tightly bonded moiré exciton,^28,29^ correlated insulator,^24,25,30^ and charge order states.^23,24,31^ Unlike the magic angle sensitivity of graphene, TMD moiré systems exhibit these strongly correlated properties in much less stringent conditions.^27,32^ Moreover, the lifted spin degeneracy due to the spin–orbit coupling also facilitates the emergence of topological phases,^33^ like valley-spin Hall insulator,^34^ quantum anomalous Hall insulator,^35,36^ and fractional quantum anomalous Hall states.^37,38^

These strongly correlated systems often involve periodic alternation in a small spatial region or undergo phase transitions in response to nanoscale environments where their properties change significantly; high spatial resolution enables the mapping of the spatial distribution of different quantum phases, helping to identify critical points and boundary regions that might not be apparent at larger scales. To enable the atomic-scale visualization of surface topography and spectroscopic measurements, scanning tunneling microscopy (STM), together with a few other real space imaging approaches,^39−41^ has been introduced to study TMD moiré systems. The unparalleled spatial resolution of STM, derived from the exponential relationship between quantum tunneling probability and the distance between the tip and a substrate, significantly empowers the direct imaging of the moiré pattern and the identification of its periodicity and phase.^42^ Scanning tunneling spectroscopy (STS) further helps to provide detailed information about the local electronic structure, allowing for the identification of flat bands and other unique electronic features. For TMD moiré superlattices on conductive substrates, STM has been instrumental in validating the formation of an unexpectedly large periodic potential,^43^ which facilitates the formation of flat bands. For TMD moiré superlattices on insulating substrates, STM, by circumventing phenomena such as Fermi level pinning and electronic screening,^44−46^ has provided a more intrinsic view of the moiré flat bands,^47^ the sensing and manipulation of electron–electron correlation,^48^ the direct observation of the 2D generalized Wigner crystal lattice in real space,^49^ and the visualization of photoexcited moiré excitons.^50^ This feature article explores the pivotal role of STM in elucidating the structural and electronic properties of TMD moiré superlattices and traces the progress of the exploration of the strongly correlated quantum phenomena hosted by these systems.

## 2 Recent STM Studies on TMD Moiré Heterostructures

### 2.1 STM Studies on TMD Moiré Heterostructures on Conductive Substrates

In 2017, Hyo Sung Kim et al. utilized STM and STS to investigate the moiré superstructure formed between ultrathin lead (Pb) films and IrTe_2_.^51^ They observed a strong lateral electronic modulation in the 2D quantum well states (QWS’s) of the Pb films with the same periodicity of the moiré structure. Specifically, in Pb islands on the hexagonal domain of IrTe_2_, the QWS’s were found to split into three distinct sub-bands, each with unique d*I*/d*V* lateral patterns and an energy splitting of approximately 120 meV. This splitting is attributed to the periodic lateral potential created by the moiré superstructure of the van der Waals heterointerface. Density functional theory (DFT) calculations further supported their statement, suggesting that the QWS splitting here is more closely linked to periodic structural distortions in the film rather than direct electronic coupling with the substrate. This study highlights the significant impact of the moiré superstructure, particularly its periodically corrugated film structure, on inducing strong electronic modulation in the overlayer film.

**Figure 1:**
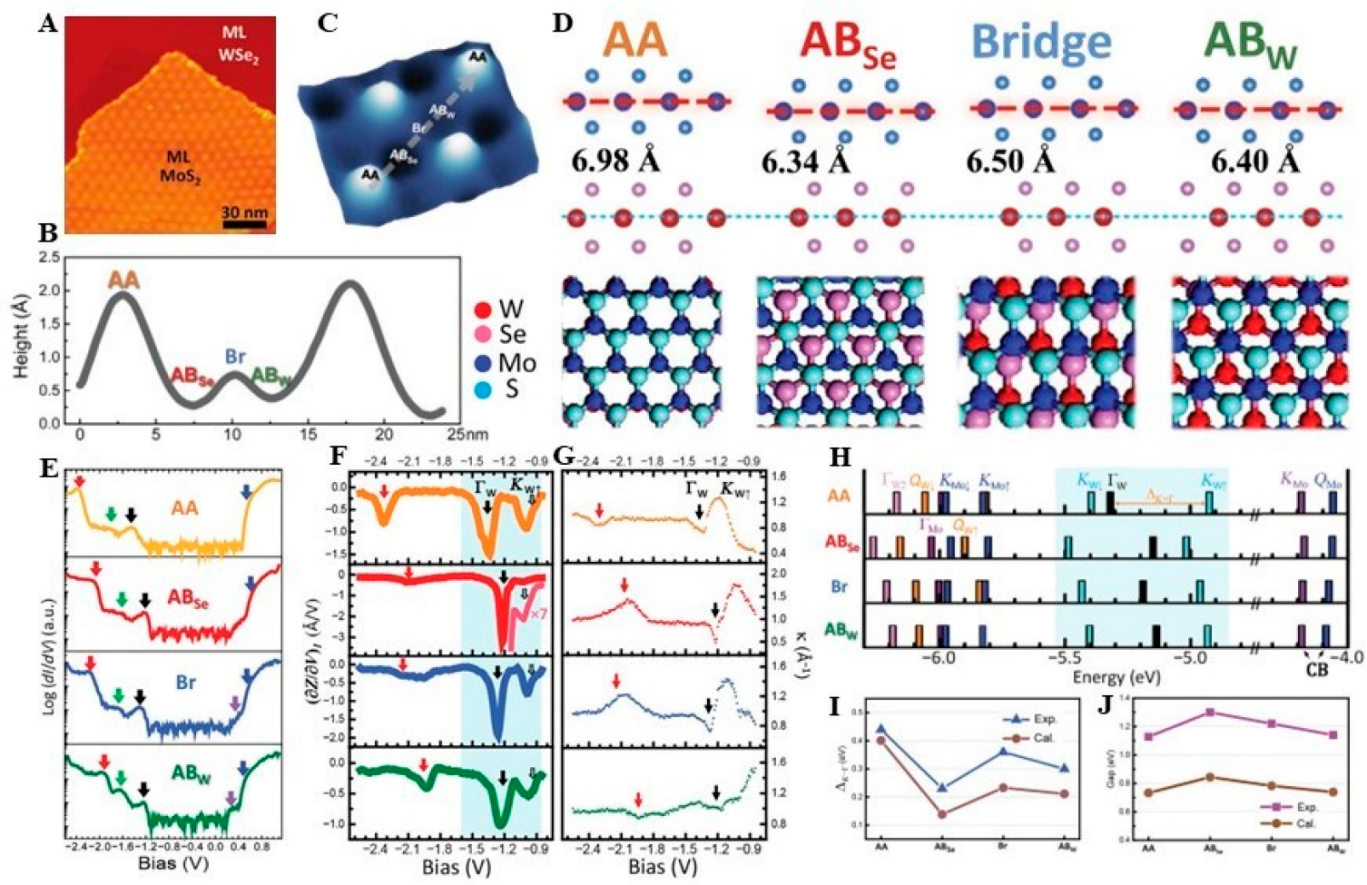
STM studies in interlayer couplings, stacking types, and 2D electronic structure in MoS_2_/WSe_2_moiré superlattice. (A) STM image of a MoS_2_/WSe_2_ van der Waals heterostructure on a highly oriented pyrolytic-graphite (HOPG) substrate. (B) Height profile from AA to AA following the corresponding direction (gray dashed line) within (C) a zoomed-in perspective of the STM image focusing on a moiré pattern unit cell. (D) Side and top views of simulated atomic models (R-type stacking) with calculated interlayer separations. (E) d*I*/d*V* spectra, (F) (∂*Z*∂*V*)_I_, (G) decay constant *k* spectra of valence bands, and (H) calculated energy values relative to the vacuum level at critical points for each site are displayed, respectively. The shaded regions in parts F and (H) represent the valence band edges and show consistent movements of the energy locations of Γ_W_ (black) and K_W_ (cyan). The spectral features marked by red and green arrows in (E) to (G) correspond to the lower energy states in (H). (I) Energy differences between K_W_ and Γ_W_ (Δ_K−Γ_) for the four atomic alignments via experiments and DFT calculations. (J) Local bandgap E_g_ between the CBM of MoS_2_ and the VBM of WSe_2_ via experiments and DFT calculations. Reproduced from ref (52) under a CC BY-NC license. Copyright 2017 The Authors.

In 2017, Chendong et al. employed STM to study the R stacking (zero-degree rotational angle) MoS_2_/WSe_2_ heterobilayers grown on graphite surface and measured STS spectra under various bias voltages.^52^ In this work, they further explore the correlations between the electronic structure, stacking configurations, and interlayer coupling in moiré superlattice. They have imaged the MoS_2_/WSe_2_ heterostructure and obtained the surface height profile via STM (Figure 1A–C), labeling four typical stacking types (AA AB_Se_ Bridge (Br), and AB_w_) with the help of first-principles calculations and corresponding atomic models (Figure 1D). First-principles calculations also predict critical points in the electronic structure, such as the K_w_ and Γ_w_ states. These critical points expected that a direct gap could be maintained in the heterobilayers, unlike homobilayers. For a deeper understanding of these critical states, Chendong et al. were then applying STS (Figure 1E). Based on the Tersoff-Hamann model, the d*I*/d*V* signal measured in STS is directly proportional to the local density of states (LDOS).^53^ To obtain a higher sensitivity than the traditional d*I*/d*V* to the states that decay fast in the *z*-direction, in this study, the authors utilized a less common approach, by keeping the feedback loop close and measuring the differential change of tip height at different bias voltages under the constant tunneling current (∂*Z*∂*V*)_I_ (Figure 1F). By comparing the d*I*/d*V* spectra and the valence bands’ (∂*Z*∂*V*)_I_ and decay constant *k* spectra (Figure 1G) for each stacking type, they observed a sudden drop in the (∂*Z*∂*V*)_I_ spectra, when the sample bias shifted from below to above calculated Γ_w_ states in the WSe_2_ layer. This observation confirmed the loss of Γ_w_ states, which arises from different lateral alignments of the S/Se’ p_*z*_ orbitals at various stacking types. This localization of Γ_w_ states was consistent with the local minimum behavior in the k spectrum because the parallel momentum *k*_||_ = 0 at Γ point and *k* = , where ϕ_0_ is the barrier height. After cohesively analyzing the behaviors on both the valence band maximum (VBM) and the conduction band minimum (CBM) on different sites (Figure 1H), they found that their DFT calculations matched well with their experimental data of the energy differences between K_W_ and Γ_W_ (Δ_K−Γ_) (Figure 1I) and the local site-dependent bandgap *E*_g_ with a periodic modulation as large as 0.2 eV, resulting in an electronic superlattice (Figure 1J). The formation of electron superlattices has been further validated by the pattern evolution in bias-dependent STM images due to electronic states at different stacking sites gradually moved out of the tunneling window as bias increasing from −3.0 V to +0.85 V.

**Figure 2:**
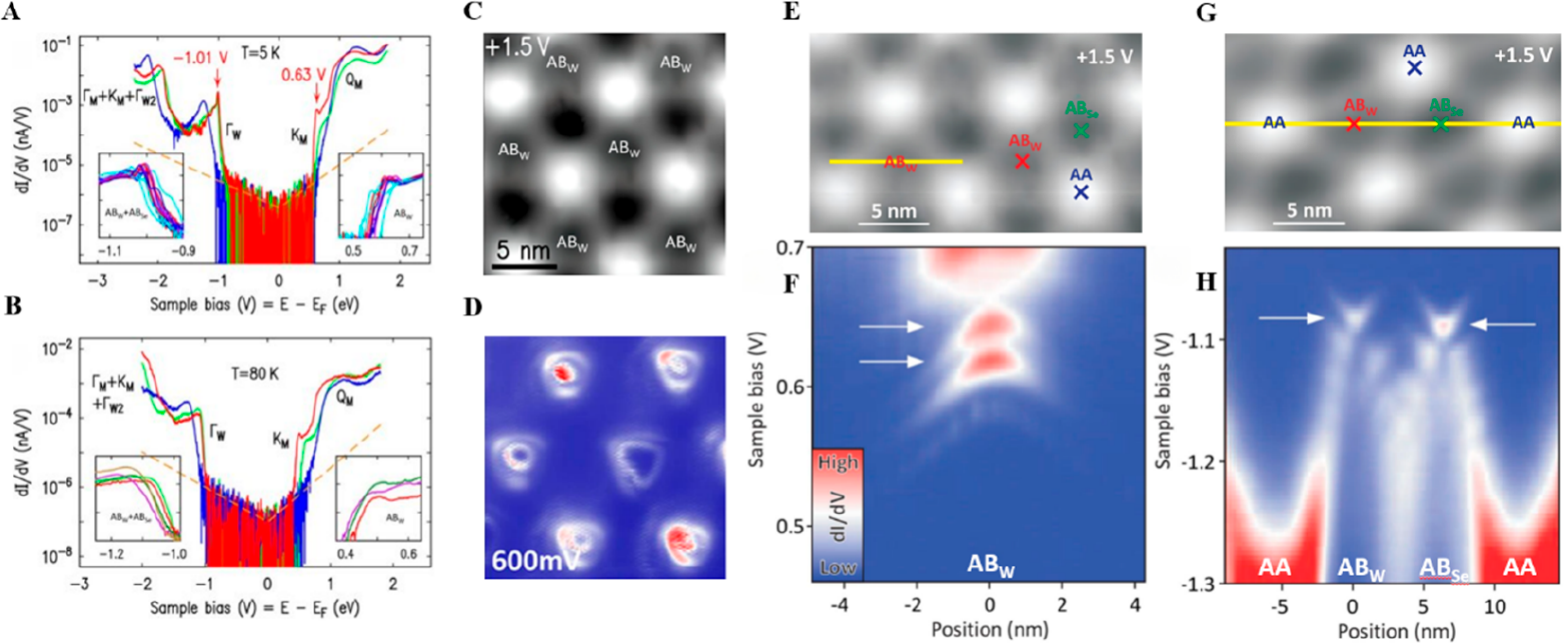
STM and temperature-dependent STS studies in quantum-confined electronic states of MoS_2_/WSe_2_ moiré heterobilayers. (A, B) STS data at 5K (A) and at 80K (B) on various local alignment sites (blue for AA, red for AB_W_, green for AB_Se_), with insets highlighting band-edge peaks and data from adjacent areas. (C) STM constant-current image of a MoS_2_/WSe_2_ heterobilayer. (D) Constant-height d*I*/d*V* map at 600 mV, showing distinct rings around AB_W_ sites, indicative of electronic states from quantum confinement effect in the moiré unit cell. (E) STM image, with moiré locations AA, AB_W_, and AB_Se_ indicated. (F) Constant-height conductance map along the yellow line in (E) at conduction band-edge voltages, showing two AB_W_-confined states (marked by arrows) and a broader AB_W_-centered dI/dV feature indicating the band onset at higher energy. (G) Similar to (E) but in a different area of the MoS_2_/WSe_2_ moiré structure. (H) Constant-height d*I*/d*V* map along the yellow line in (G) at valence band-edge voltages, showing confined states at AB_W_ and AB_Se_ (marked by arrows). Measurements from (C) to (H) are at 5 K. Reproduced with permission from ref (54). Copyright 2018 American Chemical Society.

In a following work, Yi et al. explored the electronic states in the MoS_2_/WSe_2_ moiré superlattice grown on graphene substrate at temperature of 5 K in 2018.^54^ They found that while the spectral shifts up to 0.2 eV between the maxima (AA) and minima (AB_w_ or AB_Se_) of the moiré corrugation remain, unreported sharp peaks appear in the tunneling spectra near the band edge for Γ_w_ in the valence band (VB) at AB_w_ and AB_Se_ and for K_M_ in the conduction band (CB) at AB_w_ and disappear at higher temperature (80 K) (Figure 2A, B). These peaks were attributed to quantum-confined states in the moiré unit cell, which were further demonstrated by the distinct rings around AB_w_ in the constant-height conductance map at +0.6 V (Figure 2C, D). The spatial conductance maps along the line for CB and VB are also consistent with the previous statement, where two AB_W_-confined states are observed in the CB region and confined states at AB_W_ and AB_Se_ are observed in the VB region (Figure 2E–H). To explain the origin of these spectral peaks, Yi et al. employed a nearly free electron (NFE) model on a hexagonal moiré lattice with the potential term |*V*_G_|=21 meV and derived the wave function at the Γ_w_ point, which is consistent with their experiments, though this theory only shows strong confinement of the state at AB_Se_, rather than both AB_Se_ and AB_W_ as in experiments. However, it is important to note that the MoS_2_/WSe_2_ heterostructures used in both studies were grown using chemical vapor deposition (CVD), which can present limitations in terms of sample quality as well as control over the stacking order and twist angle. Besides, the conductive substrate could largely screen the Coulomb interaction, potentially hindering the formation of strongly correlated phases.^44−46^

**Figure 3:**
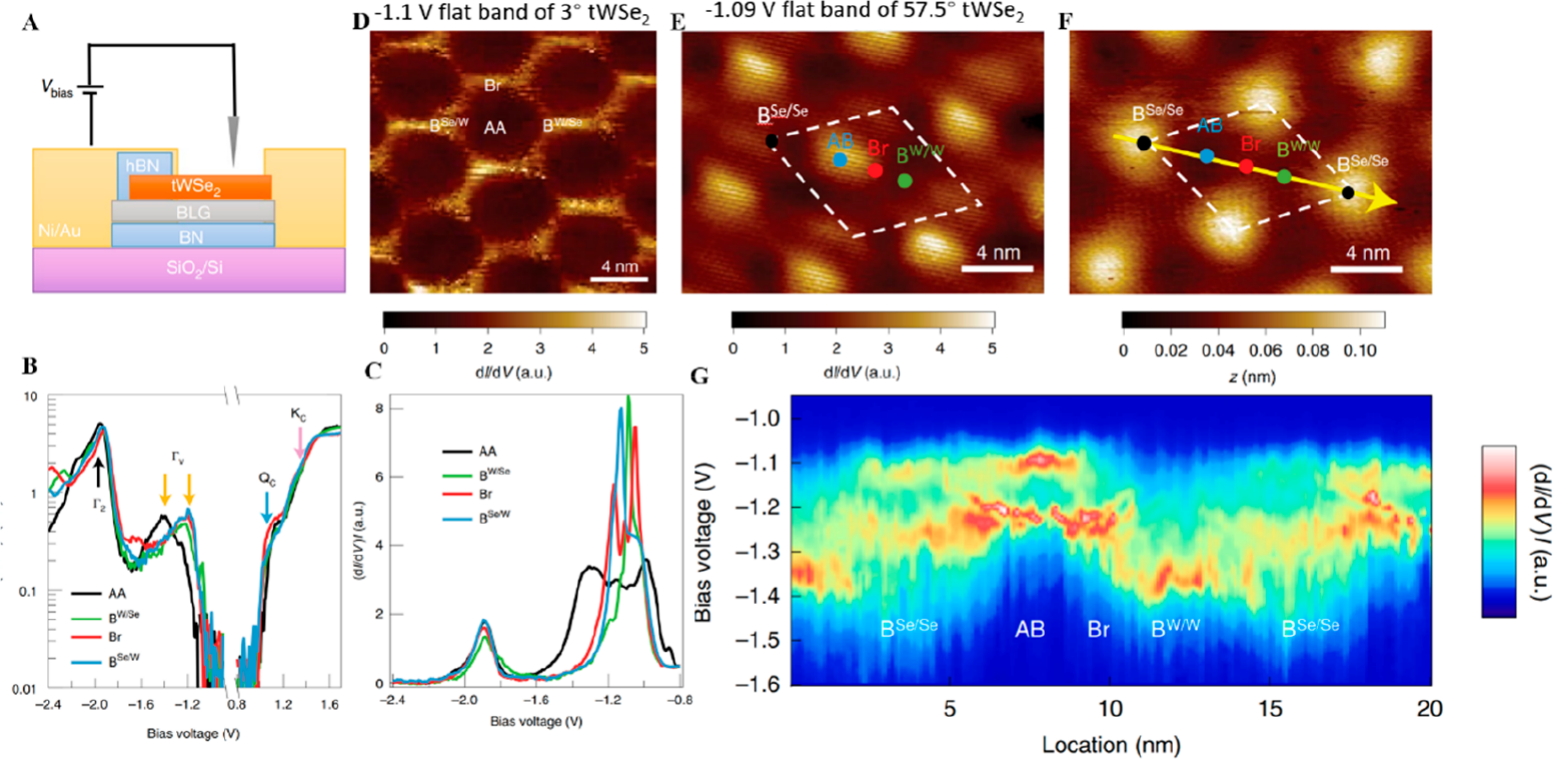
STM and STS studies in flat bands in bilayer WSe_2_with twisted angles 3° and 57.5°. (A) Schematic of the STM setup on the tWSe_2_ device. (B) Constant-height d*I*/d*V* versus bias voltage data at *I* = 100 pA, probing both the conduction and valence band states on the four stacking alignments for the 3° tWSe_2_. (C) Constant-current d*I*/d*V* versus bias voltage at *I* = 10 pA, focusing on the valence band states on different stacking alignments for the 3° tWSe_2_. (D) d*I*/d*V* map for the 3° tWSe_2_ at the flat-band energy (*V*_bias_ = −1.1 V) at *I* = 10 pA, featuring a conductive hexagon enclosing the insulating AA region. (E) d*I*/d*V* map for the 57.5° tWSe_2_ at the flat band at *V*_bias_ = −1.09 V and *I* = 50 pA. (F) STM topography on the 57.5° tWSe_2_ sample. Different stacking alignments and the unit cell are labeled in (E) and (F). (G) Constant-current d*I*/d*V* spectra along the yellow line in (F), acquired at *I* = 50 pA, indicating an isolated flat band at the AB region featured by the sharp peak around −1.1 V and multiple quantum-confined states featured by the sharp peaks between −1.2 V and −1.3 V. Reproduced with permission from ref (55). Copyright 2020 Springer Nature Ltd.

While the inversion symmetry is broken for the 0° rotation and maintains for the 60° rotation in twisted TMD, which provides an extra degree of control compared with twisted bilayer graphene, the study in the long-moiré-wavelength regime (0°< θ ≤ 7° or 52° ≤ θ < 60°) remained incomplete. In 2020, Zhiming et al. advanced the moiré electronic structure measurement of bilayer WSe_2_, prepared through the high-quality monolayers from mechanical exfoliation and specific twist angles of 3° and 57.5° from precise stacking.^55^ The superlattice was transferred onto a graphite substrate and then measured with STM and STS (Figure 3A). The authors directly measured moiré flat bands and localized states using STS and observed distinct differences between these two angles. In the case of 3° twisted bilayer WSe_2_ (tWSe_2_), the flat band was found to be localized on the hexagonal network between the AA sites, while the first flat band in 57.5° tWSe_2_ was localized on the AB sites. Constant-height STS measurements of 3° tWSe_2_ revealed bandgaps of 2.2 eV for the AA stacking and 2.1 eV for other stackings, with a valence band edge shift of approximately 80 meV (Figure 3B). Additionally, constant-current STS measurements exhibited sharp peaks at specific sites, confirming the presence of flat bands, while the LDOS maps provided further evidence of the localization of the flat band wave function (Figure 3C, D). Furthermore, in a tWSe_2_ device with a 57.5° twist angle, a distinct moiré pattern and energy bands were observed, featuring isolated flat bands and quantum-confined states (Figure 3E–G). Compared to CVD, the mechanical exfoliation approach offers enhanced control over the formation of superlattices. However, the undesirable Coulomb screening associated with conductive substrates like graphite could still hinder the formation of many strongly correlated phases.

To further elucidate the intricate relationship between twist angles and the electronic properties of TMD moiré patterns, in 2021, En et al. employed STM and STS to explore the twisted bilayer WSe_2_ on a graphite substrate.^56^ They investigated a range of twist angles, specifically 54.1°, 57°, 57.4°, 57.8°, and 58.4°, and discovered that at twist angles substantially deviating from 60°—as in the 54.1° sample—the interlayer hybridization yields only two spatially distinct flat bands with bandwidths on the order of tens of meV. However, when the twist angle approaches within 3° of 60°, lattice reconstruction occurs and, together with strong interlayer interactions, leads to the formation of triangular quantum-confined states that feature multiple energy-separated ultraflat bands with bandwidths of just a few meV—significantly smaller than the estimated on-site Coulomb repulsion energy. These experimental results are consistent with theoretical calculations and provide a groundwork for the continued exploration of correlated phases in TMD moiré systems.

### 2.2 STM Studies on TMD Moiré Heterostructures on Insulating Substrates

The increasing desire for the direct visualization of correlated quantum phases in TMD moiré superlattices has spurred a growing interest in the study of TMD moiré superlattices on insulating substrates.^57^ This will enable the tuning of charge carrier density in the superlattices by applying a gate voltage, a crucial step toward realizing many strongly correlated quantum phases. However, a major challenge arises from the low sample conductivity at cryogenic temperatures. The sample area located a few micrometers away from the contact electrode becomes insufficiently conductive for STM measurements.

**Figure 4:**
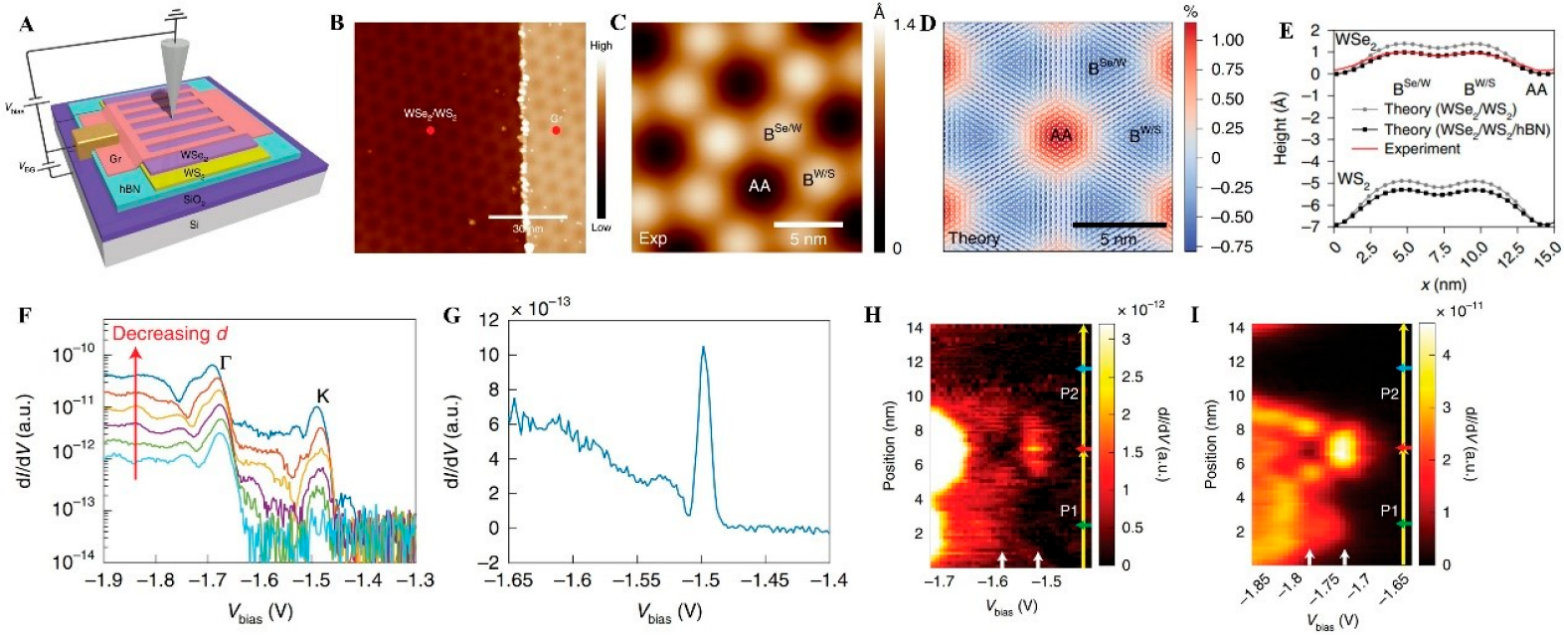
STM and STS studies in moiré flat bands in 3D reconstructed WSe2/WS2 superlattices. (A) Schematic of a gate-tunable WSe_2_/WS_2_ heterostructure device. Graphene nanoribbons (Gr) on top of the WSe_2_/WS_2_ serve as contact electrodes. (B) Ultrahigh vacuum STM image shows moiré superlattices in both the exposed WSe_2_/WS_2_ and graphene-covered area at *T* = 5.4 K, *V*_bias_ = −3 V, and *I* = 100 pA. (C) Zoomed-in STM image of the exposed WSe_2_/WS_2_ area, acquired at *V*_bias_ = −3 V and *I* = 100 pA, shows a moiré period of ∼8 nm. (D) Theoretical in-plane strain distribution (in %) for the WSe_2_ layer from simulation. (E) 3D buckling of the heterostructure via calculations and experiments. Gray and black dots show the simulated positions of W atoms for a freestanding heterostructure and a heterostructure supported by hBN, respectively. (F) Tip–sample distance(d)-dependent STS at the B^Se/W^ site, acquired at *V*_bias_ = −2.15 V and *I* = 50, 100, 200, 400, 800, 1600 pA, respectively. A second peak near *V*_bias_ = −1.5 V emerges with decreased d, indicating that it has a larger decay constant and originates from K-point states. (G) High-resolution d*I*/d*V* spectrum measured at the B^Se/W^ site. A sharp peak with fwhm of 12 ± 1 mV can be observed near *V*_bias_ = −1.5 V. (H, I) d*I*/d*V* density plot of K-point (H) and Γ-point (I) are stated in which horizontal arrows label the positions of the B^W/S^ (green), B^Se/W^ (red) and AA (blue) sites, while white vertical arrows label −1.52 V and −1.59 V in (H) and −1.73 V and −1.78 V in (I). Reproduced with permission from ref (47). Copyright 2021 Springer Nature.

**Figure 5:**
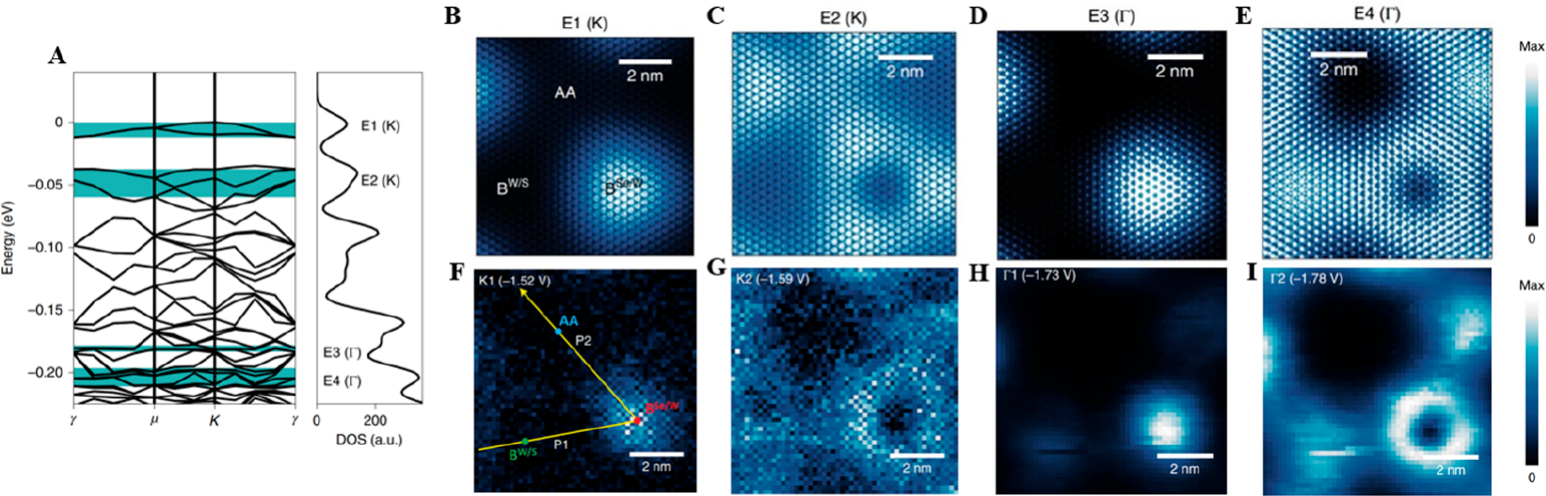
Ab initio calculations and STS measurements of the electronic structure in reconstructed moiré superlattice. (A) Electronic band structure in the folded mini-Brillouin zone via ab initio calculations (left) and the associated density of states (DOS) with 10 meV Gaussian broadening (right), featuring four key energy ranges (E1–E4) in green shaded areas. These ranges emphasize the topmost states folded from the K point (E1, E2) and Γ point (E3&E4). (B–E) Calculated LDOS maps averaged over the energy ranges labeled in (A) and over the out-of-plane direction. (F, G) Large-scale d*I*/d*V* mappings of K-point states for *V*_bias_ = −1.52 V (F) and *V*_bias_ = −1.59 V (G). (H, I) Large-scale d*I*/d*V* mappings of Γ-point states for *V*_bias_ = −1.73 V (H) and *V*_bias_ = −1.78 V (I). Panels B–I display the same sample surface area. Reproduced with permission from ref (47). Copyright 2021 Springer Nature.

In 2021, Hongyuan et al. overcame this problem and explored moiré superlattices in WSe_2_/WS_2_ heterostructures, utilizing hexagonal BN (hBN) as the electrically insulating substrate.^47^ In this study, a comb-shaped array of graphene nanoribbons was employed as a contact electrode to apply sample bias to the mechanically fabricated TMD superlattice (Figure 4A–C). The approximately 200 nm wide TMD areas situated between two adjacent graphene nanoribbons remained unaffected by the screening and maintained sufficient conductivity even at the temperature of liquid helium. Utilizing this innovative sample structure, Hongyuan et al. employed STS and ab initio simulations as investigative tools to probe and check the atomically reconstructed moiré superlattice and the consequent flat bands. Their findings revealed a pronounced 3D buckling reconstruction, referring to the out-of-plane deformation or displacement of the layers due to the created compression and tension in a moiré superstructure, and extensive in-plane strain redistribution for relaxation in the WSe_2_/WS_2_ moiré heterostructures [Figure 4(D)&(E)]. Notably, they observed a narrow, highly localized K-point (at *V*_bias_ ≈ −1.50 V) moiré flat band with a mere 10 meV width at the valence band edge of the heterostructure, in addition to other moiré flat bands originating from the Γ point (at *V*_bias_ ≈ −1.71 V) (Figure 4F, G). It is worth noticing that although both methods provide information on electronic states and properties by varying tip–sample distances, instead of employing (∂*Z*∂*V*)_I_ which is close-loop and sensitive to detect states with high decay constants, Hongyuan et al. applied tip–sample distance dependent d*I*/d*V* measurements to help determine the critical points of the moiré flat band’s electronic state. Interestingly, these observations via STS challenge pre-existing theoretical models which had predicted the AA site, rather than the B^Se/W^ site, as the localization of the 10 meV band (Figure 4H, I). To tackle this issue and better understand the observed moiré minibands, the authors conducted ab initio simulations using a calculated extensive 3D reconstructed moiré superlattice accounting for both in-plane strain and out-of-plane reconstruction (Figure 5A). While the large-scale d*I*/d*V* mappings at B^Se/W^ show the dramatical changes from LDOS maxima to minima as *V*_bias_ switching from two peak energies, −1.52 V (K1) (Figure 5F) and −1.73 V (Γ1) (Figure 5H), to slightly lower energies (Figure 5G, I), which is considered due to the delocalization of the K-point and Γ-point respectively, ab initio calculations (Figure 5B–E) match well with the experimental results correspondingly, further indicating the localization of the flat bands and proving the dominant role of 3D buckling and strain redistribution in TMD heterostructures in determining moiré electronic structure and the corresponding moiré flat bands with low electron kinetic energy. This study thus offers critical insights into the structural and electronic properties of moiré superlattices in TMD heterostructures.

**Figure 6:**
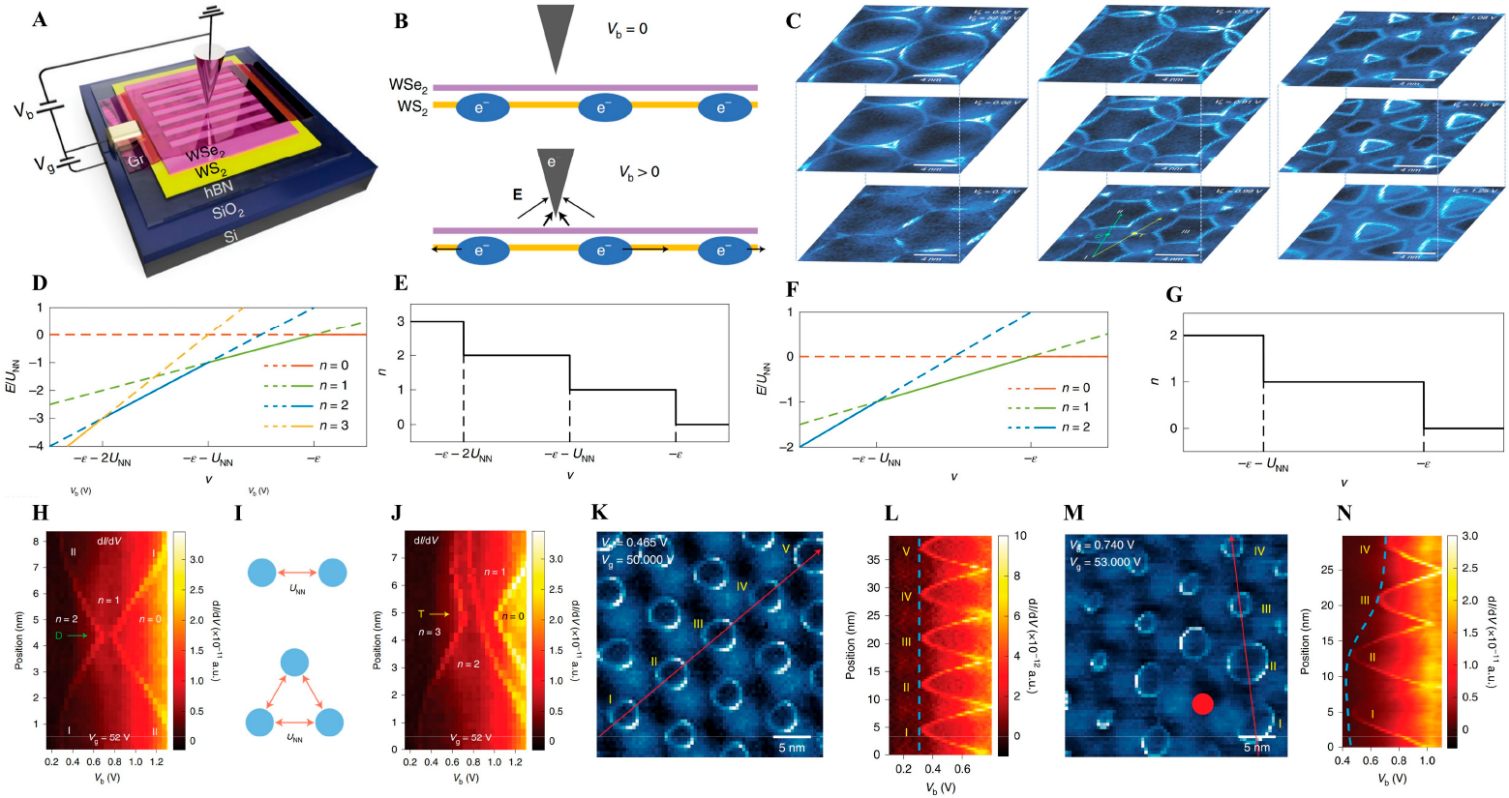
STM studies in imaging local discharge cascades for correlated electrons in WS_2_/WSe_2_ moiré superlattices. (A) Schematic of the gate-tunable WSe_2_/WS_2_ heterostructure device. Graphene nanoribbons (Gr) on top of WSe_2_/WS_2_ serve as the contact electrodes. (B) Sketch of the tip-induced moiré electron discharging. The STM tip acts as a local top gate that modifies the electron energy at nearby B^Se/W^ moiré sites and discharges them for bias voltages greater than a local threshold value. (C) Evolution of moiré discharging rings with increasing *V*_b_ at 0.57 V, 0.66 V, 0.74 V, 0.83 V, 0.91 V, 0.99 V, 1.08 V, 1.16 V, and 1.25 V, respectively, from top to bottom and left to right (*V*_g_ fixed at 52 V). The d*I*/d*V* spectra along the green and yellow lines at *V*_b_ = 0.99 V are shown in (H) and (I). Here D and T denote the two-ring and three-ring crossing points, respectively. (D, F) Calculated energy levels and (E, G) total electron number *n* of cluster ground state as a function of *v* for the three-site/two-site model, where *v* is the potential energy shift induced by *V*_b_ and *V*_g_. States with different electron numbers are labeled by color. (H) Position-dependent d*I*/d*V* spectra along the green line in (C) at *V*_b_ = 0.66 V, which passes through D. The d*I*/d*V* peaks shown by bright lines correspond to discharging events where the total electron number decreases from 2 to 1 and from 1 to 0 from left to right. Moreover, I and II indicate the moiré sites being discharged as labeled in (C) at *V*_b_ = 0.66 V. (I) Top: sketch of a simplified two-site cluster model for analysis of discharge behavior at D. Bottom: sketch of a simplified three-site cluster model for analysis of discharge behavior at T. *U*_NN_ indicates the nearest-neighbor Coulomb interaction. (J) Position-dependent d*I*/d*V* spectra along the yellow line in (C) at *V*_b_ = 0.66 V, which passes through T. Bright lines indicate discharging events at three distinct bias voltages. (K) The d*I*/d*V* map of a representative homogeneous region of the WS_2_/WSe_2_ moiré superlattice (*V*_b_ = 0.465 V, *V*_g_ = 50.000 V). (L) The d*I*/d*V* spectra measured along the red linecut shown in (K). Discharge voltages at moiré sites I–V are seen to be nearly uniform. (M) The d*I*/d*V* map of the discharge rings close to a point defect (solid red point) shows strongly nonuniform behavior (*V*_b_ = 0.740 V, *V*_g_ = 53.000 V). The defect concentration is roughly on the order of 10^10^ cm^–2^. (N) The d*I*/d*V* spectra measured along the red linecut shown in (M). A notable reduction in discharge bias is observed for sites I and II near the defect. The tip–sample distance is determined by the following mapping set points: *V*_b_ = −3 V, *I* = 100 pA. Reproduced with permission from ref (48). Copyright 2021 Springer Nature.

In addition to the quenched electron kinetic energy, the strong Coulomb interaction represents another critical characteristic meriting exploration within TMD moiré superlattices. Hongyuan et al. have delineated a STM methodology for visualizing and manipulating the charge states of correlated electrons within a gated WS_2_/WSe_2_ moiré superlattice on hBN^48^ (Figure 6A). They demonstrated that the local moiré sites’ charge states could be imaged via their impact on the STM tunneling current at different biases, similar to the phenomena previously observed near a single molecule absorbent or a localized defect. As a positive sample bias *V*_b_ exceeds a threshold value, negative charge accumulates at the tip and repels adjacent electrons, leading the localized electrons to discharge (Figure 6B). Furthermore, they experimentally successfully manipulated the charge state of correlated electrons and created a localized discharge cascade within the moiré superlattice by modulating the bias on the STM tip. They observed that the discharge rings expand with increasing *V*_b_, then starting to intersect at *V*_b_ = 0.66 V and bringing out complicated new patterns (Figure 6C). These complex patterns indicate discharging cascades due to the STM tip interactions with electrons in multiple adjacent moiré sites. Moreover, the emerging patterns deviate from the anticipated simple superposition of expanding rings predicted in a noninteracting scenario, showing the electron correlation within TMD moiré superlattices. (Figure 6(H–J). This innovative technique facilitated the determination of the nearest-neighbor Coulomb interaction (*U*_NN_) by examining the Hamiltonian of their moiré system and discerning the difference between the potential energy shifts induced by *V*_b_ and *V*_g_ at successive transition sites of the charge state of the ground state energy (Figure 6D–G). The on-site energy within the moiré superlattice was also ascertained by analyzing the spatial variation in the measured single-site discharge voltage. Specifically, at the midpoint between two or three neighboring moiré sites, a supplementary bias voltage is required to concurrently extract multiple electrons from these sites compared to the process of removing each electron individually. This additional energy penalty is the result from the intersite coulomb interaction. Therefore, by modeling the local electric field at the STM junction, Hongyuan et al. converted this bias difference into the electron–electron correlation energy. The experimental value of *U*_NN_ is determined as about 25 meV, which is about 1 order of magnitude larger than the electron bandwidth determined as about 5 meV previously calculated by DFT and a tight-binding model, warranting a platform to host strongly correlated phases. Moreover, Hongyuan et al. experimentally observed the fluctuation of moiré on-site energy due to a point defect. The comparison between a pristine region’s (Figure 6K) and a defect-affected region’s (Figure 6M) discharging behavior on the d*I*/d*V* map of the WS_2_/WSe_2_ moiré superlattice shows the nonuniformity of discharge rings caused by a point defect, which notably shifts the on-site energies of nearby moiré sites at 200 meV (Figure 6L, N). This finding helps quantitatively refine their characterization model. In general, this study has successfully showcased a versatile tool for the microscopic characterization of electron properties in materials, effectively confirming the presence of strong electron–electron correlations within TMD moiré superlattices.

**Figure 7:**
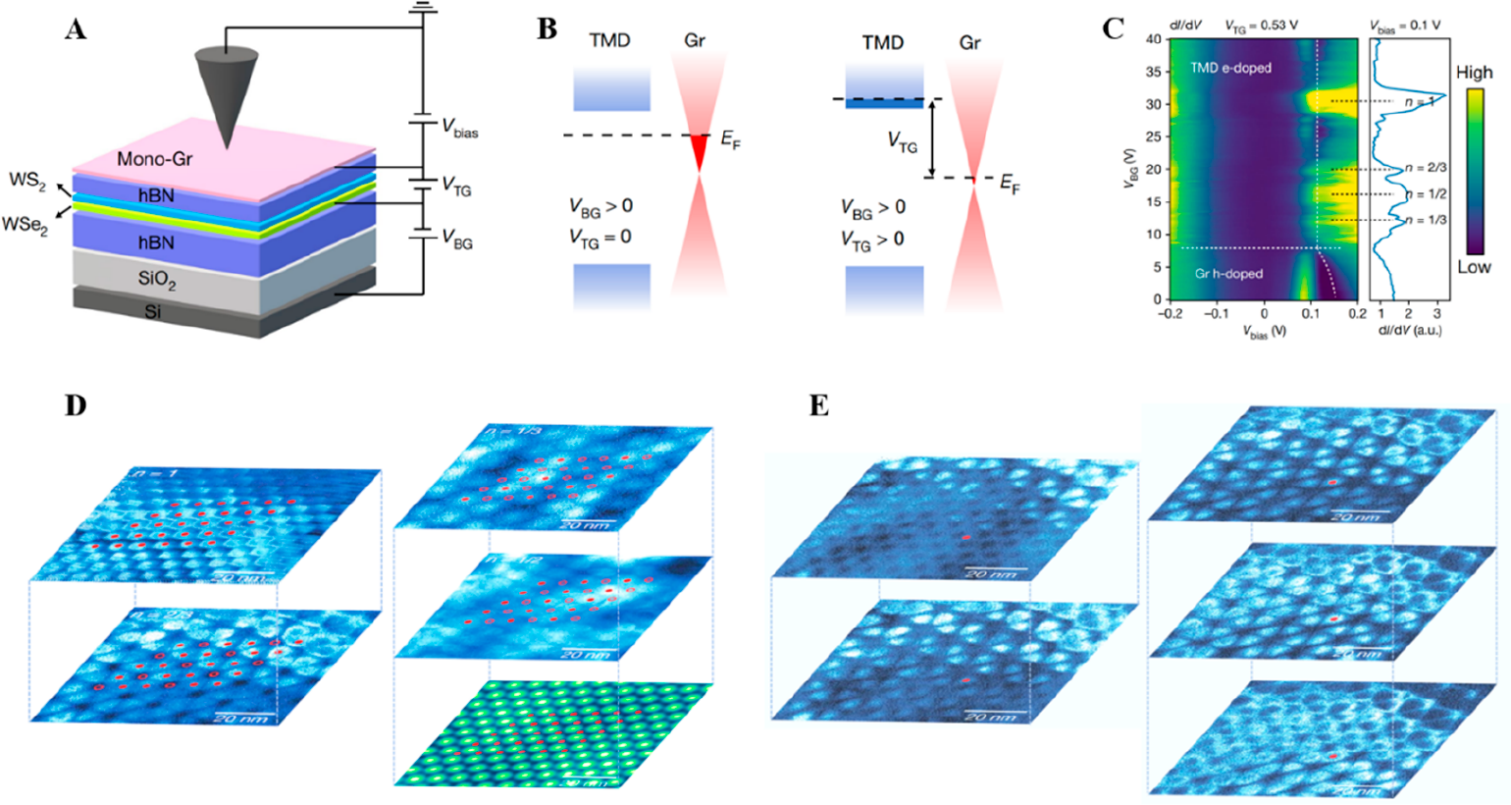
STM studies in imaging 2D generalized Wigner crystals. (A) Diagram of a dual-gated WSe_2_/WS_2_ moiré heterostructure device, featuring a top hBN layer (5 nm) slightly thinner than the moiré lattice constant (8 nm). Top gate (*V*_TG_) and bottom gate (*V*_BG_) voltages independently modulate carrier density in the WSe_2_/WS_2_ heterostructure and the top graphene sensing layer. (B) Band alignment and Fermi level schematics for V_TG_ = 0 with *V*_BG_ > 0, and *V*_TG_ > 0 with *V*_BG_ > 0, demonstrating how positive *V*_TG_ shifts the Fermi level of the heterostructure from the bandgap into the conduction band. (C) *V*_BG_-dependent dI/dV spectra on the graphene layer over an AA stacking site at *V*_TG_ = 0.53 V, showing electron doping of the graphene layer at *n* = 1/3, 1/2, 2/3, and 1. Setup: *V*_bias_ = −200 mV and *I* = 100 pA. Right graph shows a vertical line-cut of the spectra at *V*_bias_ = 0.1 V with peaks at *n* = 1, 2/3, 1/2, 1/3. (D) d*I*/d*V* maps showing: upper left, Mott insulator state of *n* = 1 (*V*_bias_ = 160 mV, *V*_BG_ = 30 V, *V*_TG_ = 0.53 V); bottom left, the generalized Wigner crystal states of *n* = 2/3 (*V*_bias_ = 160 mV, *V*_BG_ = 21.8 V, *V*_TG_ = 0.458 V); upper right, the generalized Wigner crystal states of *n* = 1/3 (*V*_bias_ = 130 mV, *V*_BG_ = 14.9 V, *V*_TG_ = 0.458 V); middle right, the generalized Wigner crystal states of *n* = 1/2 (*V*_bias_ = 125 mV, *V*_BG_ = 18.7 V, *V*_TG_ = 0.458 V); bottom right, a typical STM topographic image of the moiré superlattice without distortion or defects. Electron-filled AB_1_ sites (solid red dots) and empty AB_1_ sites (open red circles) are marked. (E) d*I*/d*V* map evolution for the *n* = 2/3 generalized Wigner crystal state under increasing *V*_bias_ (130 mV, 145 mV, 160 mV, 175 mV, and 190 mV from top to bottom and left to right), at *V*_BG_ = 21.8 V and *V*_TG_ = 0.458 V, showing a discharging ring at an electron-filled AB_1_ site (red dot) that enlarges and intensifies with increasing *V*_bias_. Reproduced with permission from ref (49). Copyright 2021 Springer Nature.

Based on the research referenced earlier, it becomes evident that within the isolated WS_2_/WSe_2_ moiré superlattice the prevailing influence of long-range Coulomb interaction energy outweighs the effects of quantum fluctuations on electron motion. Consequently, this scenario leads to the anticipation of the emergence of a predicted state known as the generalized Wigner crystal state—an orderly arrangement resembling a crystalline structure formed by electrons.^24,58−60^ However, the direct observation of the 2D Wigner crystal lattice in real space has remained a challenge. Conventional STM, despite its high spatial resolution, can induce perturbations in the semiconducting samples due to the tip-induced band-bending referenced earlier. This could significantly affect the delicate 2D generalized Wigner crystal lattice during experimentation. To address this issue, Hongyuan et al. developed a noninvasive STM spectroscopy technique in 2021, utilizing a graphene sensing layer over the WSe_2_/WS_2_ moiré superlattice.^49^ In their experimental design, the carrier densities in the WSe_2_/WS_2_ moiré superlattice and the top graphene sensing layer were controlled by the top gate voltage (*V*_TG_) and bottom gate voltage (*V*_BG_) in their setup (Figure 7A). They determined that a *V*_TG_ of approximately 0.5 V is optimal as it elevates the WSe_2_/WS_2_ heterostructure Fermi level near the conduction band edge while maintaining the graphene sensing layer close to charge neutrality (Figure 7B). This balance allowed for higher sensitivity in imaging Wigner crystal states and minimized the screening effect on the moiré electron–electron interactions. In their experiments, they observed that the graphene layer underwent electron doping when the WSe_2_/WS_2_ moiré superlattice exhibited fractional filling of *n* = 1/3, 1/2, 2/3 at the AA stacking site with *V*_BG_ > 7 V and *V*_TG_ = 0.53 V (Figure 7C). This observation indicated the presence of correlated gaps in the corresponding states in the heterostructure, rendering the WSe_2_/WS_2_ heterostructure electronically incompressible and forcing electrons into the graphene sensing layer. Utilizing this principle, they employed 2D d*I*/d*V* mapping of the graphene sensing layer to image the 2D electron lattice of the correlated insulating states in real space. The tunnel current between the STM tip and the graphene varies depending on the charge state of the detected moiré site. They observed a honeycomb lattice under *n* = 2/3, a triangular lattice under *n* = 1/3, and a stripe phase under *n* = 1/2 (Figure 7D). To further investigate their imaging method, they examined the evolution of the *n* = 2/3 d*I*/d*V* map with increasing bias voltage (*V*_bias_) and constant gate voltages. As *V*_bias_ increased, the AB_1_ stacking site became brighter, forming ring-like features, similar to the behavior observed under tip-induced electrical discharging (Figure 7E). This result suggests that the imaging of Wigner crystal lattices in d*I*/d*V* maps is enabled by the discharging of the moiré electron beneath the tip when *V*_bias_ exceeds a threshold value. This study lays a foundation for understanding Wigner crystal states in moiré heterostructures and proposes a general approach for imaging novel correlated electron lattices in other systems.

**Figure 8:**
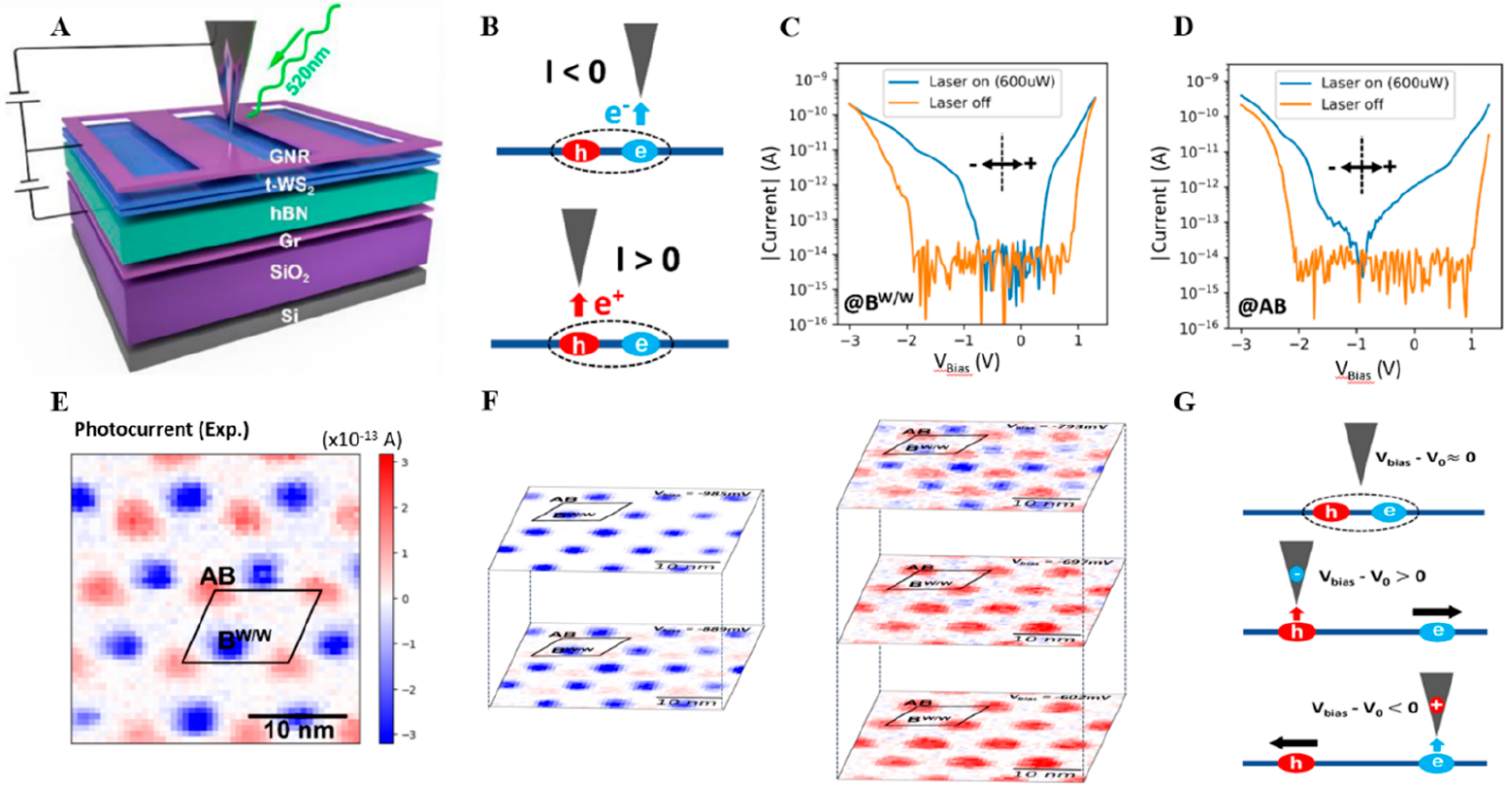
STM studies in imaging moiré excited states with PTM. (A) Schematic of the laser-STM setup for a near-58-degree twisted bilayer WS_2_ (t-WS_2_) device. The t-WS_2_ is layered over 49 nm thick hBN and a graphite substrate, serving as the gate dielectric and back gate, respectively. A back gate voltage of *V*_BG_ is applied between the t-WS_2_ and the graphite. A graphene nanoribbon (GNR) array on t-WS_2_ serves as the contact electrode. A sample-tip bias *V*_bias_ is applied between the t-WS_2_ and the STM tip to induce a tunnel current, while a 520 nm wavelength continuous-wave laser focuses on the tip-tunnel junction. (B) Schematic of tip-position dependent tunnel current from an ICT exciton. Negative current results when the STM tip is above the electron (top panel), while positive current is detected above the hole (bottom panel). (C, D) STM tunnel current spectra at the (C) B^W/W^ and (D) AB stacking sites with the laser off (orange) and on (blue) at *V*_BG_ = 0. With the laser off, the current at both sites reflects an energy gap for −2 V < *V*_bias_< 1 V. With the laser on (*P* = 600 μW), photocurrent appears in this gap, showing different photocurrent spectral shapes at B^W/W^ and AB sites. (E) A photocurrent map of t-WS_2_ with the laser on (*P* = 600 μW) at *V*_bias_ = −0.60 V and *V*_BG_ = 0 shows positive (negative) photocurrent at the AB (B^W/W^) sites. (F) Evolution of t-WS_2_ photocurrent maps for increasing *V*_bias_ at −985 mV, −889 mV, −793 mV, −697 mV, and −602 mV, respectively, from top to bottom and left to right, showing alternating current polarity in an ∼200 mV *V*_bias_ range, with negative (positive) current dominates in the lowest (highest) *V*_bias_. (G) Diagrams of the tip-induced ICT exciton dissociation effect, where *V*_0_ is the bias voltage offset that compensates the work function difference between the tip and the back gate graphite. For *V*_bias_ – *V*_0_ ≈ 0, the tip minimally affects the ICT exciton; hence, both photocurrent polarities are seen. For *V*_bias_ – *V*_0_ > 0 (*V*_bias_ – *V*_0_ < 0), negative (positive) charge accumulates at the tip apex and repels electrons (holes), thereby dissociating ICT excitons. Reproduced with permission from ref (50). Copyright 2024 Springer Nature.

In addition to solely relying on the tunneling electron to probe material properties, combining STM with optical excitation has also spurred the investigation of the moiré quantum phenomena related to light-matter coupling. In 2023, Hongyuan et al. introduced a pioneering method termed photocurrent tunneling microscopy (PTM).^50^ This technique was devised to directly visualize the electron and hole distribution within the photoexcited in-plane charge-transfer (ICT) moiré exciton in twisted bilayer WS_2_ (t-WS_2_). This technique was achieved by integrating laser excitation with an STM (Figure 8A). Originating from the competition between the electron–hole Coulomb interaction and the moiré potential landscape, ICT moiré excitons own a special characteristic of producing opposing tunneling currents based on the tip’s position over the exciton (Figure 8B). Utilizing this property, Hongyuan et al. constructed a photocurrent map of t-WS_2_ under specific conditions with a laser power of 600 μW, a bias voltage (*V*_bias_) of −0.60 V, and a bottom gate voltage (*V*_BG_) of 0 V (Figure 8C, D). This map revealed positive photocurrents at AB sites and negative photocurrents at B^W/W^ sites, providing experimental evidence for the presence of ICT moiré excitons (Figure 8E). Furthermore, both computational and experimental data presented by Hongyuan et al. indicated that the bias voltage range for the coexistence of positive photocurrent at AB sites and negative photocurrent at B^W/W^ sites is approximately 200 mV (Figure 8F), which implies that for *V*_bias_ either larger or smaller than *V*_0_, where V_0_ represents the bias voltage offset of the work function difference between the tip and the back gate graphite, there would be a tip-induced exciton dissociation (Figure 8G). The development of the PTM technique for the first time enables imaging of ICT moiré excitons with subnanometer resolution.

## 3 Conclusion and Outlook

In this paper, we have discussed the development of STM studies on imaging and characterizing TMD moiré superlattices. This evolution has significantly expanded our understanding of these intricate systems through continuous innovation of device design, integration of photon excitation with STM, and meticulous research. Initially, the samples that are suitable for the STM study were predominantly grown using chemical vapor deposition (CVD) on graphite substrates. This method facilitated the observation of electronic band structure influenced by the periodic moiré potential.^52^ Notably, evident quantum-confined states near the band edge at stacking configurations AB^w^ and AB^Se^ were identified, corroborated by constant-height conductance maps and the NFE model.^54^ However, CVD-grown samples presented challenges, including compromised sample quality and limited control over stacking order and twist angle. The use of conductive substrates further introduced strong Coulomb screening, impeding the exploration of strongly correlated phases.^44−46^ Another crucial disadvantage for conductive substrates is that it makes gating samples and modulating carrier densities in device difficult, which has been proven, latterly, as shown in the last section, to be significant in studying electronic states and properties of TMD moiré superstructures under different situations.

To enhance sample quality, the focus shifted to mechanically exfoliated samples, offering superior control over TMD moiré pattern formation. These samples elucidated variations in the electronic structures of TMD superlattices attributable to different twist angles. Initially, the exfoliated sample was transferred to a graphene substrate.^55^ Subsequently, after employing insulating substrates such as hBN, to address the conductivity challenges of TMD heterostructures on insulating substrates at low temperatures, researchers modulated the electron/hole concentration through bottom gate voltage. Additionally, graphene nanoribbons were incorporated as contact electrodes on top of the samples. Combining both mechanical exfoliation approach and insulating substrates, this innovative design facilitated the examination of strain redistribution and 3D buckling in TMD heterostructures, confirming the presence of strongly correlated flat bands with low electron kinetic energy.^47^ Taking advantage of the strong Coulomb interaction within TMD superlattices, this new device also enabled the studying and manipulation of intrinsic strongly correlated phenomena, like correlated electron charge states, by adjusting the STM tip bias.^48^

Given the inherent properties of TMD moiré superlattices, including quenched electron kinetic energy and strong electron–electron correlations, the potential emergence of a generalized Wigner crystal state was expected. However, perturbations from the STM tip presented observational challenges. To get rid of such perturbations, a graphene sensing layer was introduced atop the TMD moiré pattern.^49^ This layer facilitated electron transfer to graphene when the TMD heterostructure exhibited electronic incompressibility under certain fractional fillings, and the bias voltage surpassed a threshold. Beyond mere tunneling electrons, optical excitations have also garnered interest in STM studies. A prime example is the measurement of photocurrent, highlighting advancements in visualizing photoexcited in-plane charge-transfer moiré excitons in TMD heterostructures by probing the opposing tunneling current across exciton sites.^50^

Apart from the previously mentioned correlated phenomena, STM shows a promising future in exploring other correlated features of moiré quantum phases as well, such as the correlated insulator state^24,25,61^ and superconductivity^62−64^ in TMD moiré materials. For example, the charge-transfer insulator state emerges in multiple TMD materials when on-site Coulomb repulsion dominates electron kinetic energy at one hole per superlattice site (corresponding to half-band filling).^65,66^ This state corresponds to the freezing of the charge degree of freedom, allowing electrons only to move between anion-like and cation-like orbitals within the unit cell, to reduce on-site Coulomb repulsion.^64^ Meanwhile, collective spin excitations from local magnetic moments dictate the low-energy dynamics. This phenomenon has been corroborated through temperature-dependent magnetic circular dichroism (MCD) measurements, taking advantage of the spin-valley-locked band structure and the valley-dependent optical selection rules inherent to monolayer TMDs.^21,33,65,67^ Additionally, the spin relaxation lifetime in the charge-transfer insulator state was found to be significantly longer than that of charge excitations and further studies are needed to investigate how the persistent spin excitations from the charge-transfer insulator state can elucidate its spin configuration.^24^ Aiming to reduce the overall Coulomb repulsion in TMD heterostructures at half-filling, doping with holes results in the formation of tightly bound charge-2e excitations, known as trimers, which comprise a pair of holes bound to a charge-transfer exciton.^64^ When the bandwidth of doped holes is small, the trimers form insulating pair density waves at specific doping levels, denoted as *n* = 1 + *p*/*q* > 1, where *p* and *q* are integers, with periodicity in alignment with the moiré lattice. As the bandwidth approximates the pair binding energy, a resonant interaction occurs between itinerant holes and charge-2e trimers, leading to unconventional superconductivity. The complexity and intrigue surrounding these correlated electron phenomena necessitate a microscopic understanding, underscoring the significant potential of STM in advancing future research.

In addition to the demonstrated successes in hard condensed matter phases, TMD moiré systems also hold significant potential in the field of chemistry,^66,68−70^ particularly concerning charge transfer behavior. For example, recent studies have observed modification on the chemical reactivity of the moiré heterostructures, which is attributed to the interplay between the moiré potential and Coulomb interactions. In the case of WSe_2_/WS_2_ heterostructures with a 3° twist angle, STS detected charge transfer occurring over distances on the order of 10 nm, from MM to MX spots, which is considered as a consequence of the increasing filling factors and the resulting rise in repulsive interaction particularly in the context of charge transfer behavior.^66^ Moreover, the twist angle itself has been identified as a critical variable in modulating charge transfer kinetics within moiré patterns.^69^ In the case of twisted bilayer graphene with a twist angle below 5°, the observed intrinsic electron transfer rate at AA sites, where flat bands are localized, significantly exceeds predictions made by the Gerischer–Marcus model. This significant local electrochemical enhancement is optimized near the magic angle of 1.1° and is attributed to the presence of moiré-derived highly localized flat bands and the structural relaxation of the moiré superlattice.

Looking ahead, we foresee that manipulating the moiré potential will emerge as a central strategy for both manipulating the electron correlated phenomena and enhancing chemical reactivity. Notably, many of these phenomena remain unexplored at the nanoscale, signaling the existence of a vast research frontier that has yet to be traversed. In this endeavor, the STM is poised to be an invaluable tool for navigating these uncharted waters with unparalleled spatial visibility.
